# Temporal dynamics of students’ physiological arousal in personalized learning contexts: a quasi-experimental study based on electrodermal activity

**DOI:** 10.3389/fpsyg.2026.1872311

**Published:** 2026-08-24

**Authors:** Xiangying Xue, Xiangcong Liu, Yuqi Dong, Xingye Chen, Yuanqing Pei

**Affiliations:** 1 School of Communication, Qufu Normal University, Rizhao, China; 2 School of Education, Shanghai Normal University, Shanghai, China; 3 Shanghai Experimental School, Shanghai, China; 4 Shanghai Experimental Primary School, Shanghai, China

**Keywords:** academic emotion, cognitive state, electrodermal activity, personalized learning, physiological arousal

## Abstract

Physiological arousal varies throughout classroom learning, yet its temporal dynamics during personalized learning in authentic primary-school classrooms remain unclear. This quasi-experimental study used electrodermal activity (EDA) to examine the lesson-wide temporal trend, dynamic trajectory, and phase-specific arousal level and activation frequency in an “Introduction to Circles” lesson. Two intact fourth-grade classes in Shanghai participated (analytic sample: experimental *n* = 23; control *n* = 27). The experimental class received personalized learning based on cognitive diagnosis, targeting discrepancies between students’ existing conceptions and target concepts; the control class received conventional instruction. Raw skin conductance (SC) and skin conductance response frequency (SCRF) served as operational indicators; within-person min–max standardized SC (SSC) served as a robustness check. Mann–Kendall tests, individual-level linear mixed-effects models, and matched-time comparisons were conducted. Across 17 two-minute windows, SSC declined significantly in both classes (control: *Z* = −2.266, *p* = 0.023; experimental: *Z* = −4.490, *p* < 0.001). The raw-SC model showed a significant Condition × Time interaction at the two-minute resolution (*p* = 0.008), but not at four or six minutes. Of four event-boundary adjacent-window comparisons, two Condition × Phase interactions remained significant after Holm correction: SC increased in the experimental class and decreased in the control class at the first episode start (adjusted *p* = 0.012); the pattern reversed at the second episode end (adjusted *p* = 0.002); both were reproduced using SSC. Phase-average raw SC did not differ between conditions (*p* = 0.900), whereas SCRF was higher in the experimental class (*p* < 0.001, *d* = 2.76). Post-test mathematics achievement was also higher in the experimental class (*p* = 0.005). Physiological arousal declined as the lesson progressed; the personalized-learning condition did not consistently alter this trajectory but the two classes showed different short-term SC changes at two instructional transitions. The higher post-test achievement suggests a potential learning benefit; however, the relationship between these physiological patterns and the achievement difference warrants further investigation.

## 1 Introduction

Academic emotions—the emotions students experience in achievement-related contexts—substantially influence cognitive engagement, self-regulated learning, and academic outcomes (Pekrun, 2006; Pekrun et al., 2002). Within the framework of the Control-Value Theory (CVT), Pekrun (2006) posits that students’ appraisals of their control over learning tasks and their subjective evaluations of learning outcomes elicit discrete emotional states, which in turn shape their motivational and cognitive processes. A substantial body of empirical research broadly supports this theoretical account: positive activating emotions (e.g., enjoyment and curiosity) facilitate deep processing and sustained effort, whereas negative deactivating emotions (e.g., boredom) impair cognitive engagement and academic performance (D’Mello, 2013; Loderer et al., 2020). Emotions are therefore not peripheral to instruction; as Graesser (2020) aptly notes, emotions serve as the experiential glue that binds learning environments together.

Academic emotions are widely conceptualized as multi-component constructs comprising three components: subjective feeling, expressive behavior, and physiological arousal (Izard, 1991; Hockenbury and Hockenbury, 2007). Most existing research relies on self-report instruments to capture students’ emotional states. While such instruments are well suited for assessing the subjective experiential component, they are subject to retrospective bias, social desirability effects, and the practical difficulty of repeatedly interrupting ongoing learning to administer measures (Noroozi et al., 2020). The expressive component, typically captured through facial expression analysis or body posture coding, has also been explored in educational research, but its application in authentic classroom settings is constrained by occlusion (e.g., students looking down at materials), inter-rater reliability concerns, and the substantial labor required for behavioral coding (Hu and Gao, 2025; Lyu et al., 2022). In contrast, physiological measures can continuously record changes in physiological arousal without disrupting the natural instructional flow. This advantage motivates the present study’s use of electrodermal activity (EDA). However, EDA alone cannot identify a specific academic emotion, its valence, or the psychological process responsible for the observed activation.

Despite increasing attention to physiological measurement in educational research, the application of EDA in authentic classroom contexts remains limited. This limitation reflects several practical and methodological challenges, such as the complexity of conducting synchronized multi-participant data acquisition, the demands of large-scale signal processing, and the difficulty of mitigating motion artifacts inherent to active classroom participation—challenges that are easier to address in controlled laboratory environments or short-duration technology-mediated tasks. Existing studies have predominantly examined EDA in controlled laboratory environments or technology-mediated tasks (Horvers et al., 2024; Huber and Bannert, 2023; Lal et al., 2024; Zheng et al., 2024), with comparatively little attention paid to how physiological arousal unfolds throughout authentic classroom instruction. More fundamentally, the temporal structure of arousal dynamics—that is, how arousal levels fluctuate in response to discrete instructional events—has rarely been subjected to systematic empirical examination. Although it is important to understand whether arousal differs across instructional conditions, a more fundamental question concerns when and how arousal responds to specific instructional moments. This question represents a substantive gap in the current literature.

Personalized learning, an important instructional approach in educational technology research, is characterized by the alignment of learning tasks, pace, and support structures with learners’ prior knowledge and cognitive readiness (Kim, 2012). Beyond its well-documented cognitive effects, personalized learning is also theoretically expected to influence students’ affective engagement, although different theoretical perspectives offer different accounts of this influence. For example, from the perspective of the Control-Value Theory, personalized learning may enhance students’ sense of control over learning tasks by matching instructional demands with learners’ existing competence—a core antecedent of positive academic emotions within the CVT framework (Pekrun, 2006; Daniels and Stupnisky, 2012). The present study examines a specific form of personalized learning guided by cognitive diagnosis, in which discrepancies between students’ diagnosed conceptions and scientifically accurate representations are presented during instruction (hereafter, conflict-based personalized learning). These discrepancies may be accompanied by sympathetic activation associated with an orienting response, but physiological activation may also reflect cognitive effort, uncertainty, or anxiety. Consequently, EDA cannot by itself establish that students experienced positively valenced emotions or that cognitive conflict was the psychological source of the activation. However, empirical research on how this specific form of personalized learning is associated with students’ physiological arousal in classroom settings remains scarce. Whether this instructional condition is associated with distinctive arousal patterns relative to conventional classroom instruction—and whether such differences manifest in overall arousal levels, activation frequency, or temporal trajectories—remains unresolved. In particular, its physiological arousal patterns remain under-examined in authentic primary-school classrooms, constituting the research gap addressed in this study.

The present study addresses this gap through a quasi-experimental investigation conducted in an authentic primary-school classroom. Focusing on physiological arousal during academic activity, this study uses raw skin conductance (SC) and skin conductance response frequency (SCRF) as operational indicators to examine whether the personalized-learning condition is associated with distinctive arousal characteristics across three dimensions: the overall temporal trend across the lesson, the dynamic trajectory of arousal, and arousal level and activation frequency during the personalized-learning phase. The SC analyses were repeated using within-person min–max standardized skin conductance (SSC) to assess whether the findings were robust to individual differences in absolute SC levels and ranges.

*RQ1*: Do students under conflict-based personalized learning and conventional classroom instruction exhibit differing temporal trends of physiological arousal across the lesson?

*RQ2*: Do the two groups exhibit significant differences in the dynamic trajectory of physiological arousal, and do the observed differences correspond temporally to specific instructional events?

*RQ3*: Compared with conventional classroom instruction, do students under conflict-based personalized learning exhibit significantly different physiological arousal levels and activation frequencies during the personalized learning phase?

## 2 Literature review

### 2.1 Academic emotions and physiological arousal in learning

Within the scholarly tradition of the Control-Value Theory (CVT; Pekrun, 2006), a substantial body of research has documented systematic associations between students’ habitual emotional dispositions (e.g., enjoyment, anxiety, and boredom) and their cognitive engagement, self-regulation, and academic achievement (Pekrun et al., 2002; Pekrun and Linnenbrink-Garcia, 2012; Goetz et al., 2006). The mainstream research approach within this tradition, however, has tended to characterize academic emotions as relatively stable trait-like dispositions, typically measured through retrospective self-report instruments that aggregate emotional experiences over extended time periods. Recent research has increasingly emphasized that academic emotions also unfold dynamically and in real time during instruction, with moment-to-moment fluctuations in emotional states shaping students’ immediate cognitive engagement in ways that aggregated trait measures cannot capture (Lo, 2024). Consequently, a growing body of research has shifted toward state-level emotional dynamics, examining how emotional states emerge, evolve, and dissipate within specific learning episodes (D’Mello, 2013; Graesser, 2020; Camacho-Morles et al., 2019).

This shift toward state-level emotional dynamics carries important implications for clarifying which emotional dimension is most consequential during the learning process. Trait-level approaches naturally emphasize the valence dimension—focusing on whether students habitually feel positive or negative toward academic activities. State-level approaches, in contrast, foreground the moment-to-moment intensity of emotional activation, directing researchers’ attention to the arousal dimension. Storbeck and Clore (2008) argue that arousal is not merely a concomitant of emotional valence but rather embodied information about the importance and urgency of current cognitive content—amplifying engagement with ongoing processing strategies, enhancing attention to salient stimuli, and facilitating long-term memory consolidation. Two emotional states of identical valence may therefore differ substantially in arousal: curiosity and contentment are both positive emotions, yet they may differ in the degree of sympathetic activation; elevated arousal may also accompany negatively valenced states such as anxiety. Capturing this arousal dimension at the temporal resolution required for state-level research necessitates objective and continuous measurement, for which electrodermal activity (EDA) has become the most widely adopted physiological tool in educational research (Caruelle et al., 2019; Posada-Quintero and Chon, 2020). EDA comprises two analytically distinguishable components: a tonic component reflecting background sympathetic activation level and a phasic component reflecting event-locked sympathetic responses to specific stimuli (Boucsein et al., 2012). This dual structure enables the simultaneous examination of overall arousal states and discrete activation moments in temporal relation to specific learning events. Nevertheless, EDA indicates the intensity of physiological activation but cannot by itself determine emotional valence or identify the specific psychological process responsible for that activation. In authentic classrooms, phasic responses may also be influenced by movement and device interaction.

A growing body of empirical research has applied EDA to the investigation of physiological arousal during learning (Järvelä et al., 2021). The systematic review by Horvers et al. (2021) surveyed the application of EDA in educational contexts, noting that EDA is sensitive to physiological activation during learning, although methodological practices vary substantially across studies. Ketonen et al. (2023) demonstrated a positive association between students’ self-reported excitement and physiological arousal during learning tasks, illustrating correspondence between subjective reports and physiological activation in that study. Yu et al. (2024) found that dynamic changes in EDA were significantly associated with students’ learning efficiency in university classroom settings, suggesting that EDA may serve as a valuable physiological indicator for predicting classroom performance. Lal et al. (2024) jointly examined physiological and gaze-based indicators of learner emotions, illustrating the value of combining EDA with other measures when investigating learner emotions. Horvers et al. (2024) further examined EDA peaks in relation to students’ responses to immediate feedback in an adaptive-learning context. Despite these advances, three notable limitations persist in the existing literature. First, most EDA studies have been conducted in controlled laboratory environments or technology-mediated tasks rather than authentic classroom contexts. Second, existing research primarily compares arousal levels across conditions, paying limited attention to the fine-grained temporal structure of arousal dynamics within a lesson. Third, the correspondence between physiological arousal transitions and discrete instructional events has rarely been examined systematically. The present study addresses these limitations by analyzing continuous EDA data collected throughout an entire classroom lesson, focusing on the overall temporal trend, the dynamic trajectory of arousal, and the temporal correspondence between observed changes and specific instructional events. Physiological monitoring has also been reviewed as an input to intelligent and adaptive learning environments (Lane and D’Mello, 2018; Goussain et al., 2025). However, this literature has largely emphasized technology-mediated systems and broad application domains rather than event-associated SC changes during a complete authentic primary-school lesson.

### 2.2 Personalized learning: effects and mechanisms

Research on personalized learning has developed along multiple methodological lines, including adaptive learning systems that dynamically adjust task difficulty (Aleven et al., 2017), interest- and context-based personalization that aligns instructional content with students’ personal interests (Walkington, 2013), and learning-analytics-driven recommendation-based personalization (Hsu et al., 2013). Recent reviews and meta-analyses have strengthened the empirical basis of personalized learning. In a scoping review covering 69 studies from 2012 to 2024, Du Plooy et al. (2024) reported robust positive effects of personalized adaptive learning on academic performance and engagement. Wang et al. (2024) corroborated this conclusion in their meta-analysis of adaptive learning systems from 2010 to 2022. Hu (2024), drawing on a meta-analysis of 31 empirical studies, found that AI-assisted personalized learning produces a medium-to-large effect on cognitive learning outcomes (*g* = 0.70). These reviews converge on a core conclusion: personalization is most effective when it responds to substantive learner characteristics—particularly cognitive states—rather than superficial features (Bernacki et al., 2021).

The personalized learning approach adopted in the present study belongs to the cognitive-diagnostic tradition, which focuses on identifying students’ specific prior conceptions and misconceptions as the basis for personalized instruction (Duit and Treagust, 2003; Treagust, 1988). Specifically, this study employs the two-tier cognitive state framework developed by Dong and colleagues (Dong et al., 2023). In this framework, a “cognitive state” refers to a learner’s pre-instructional understanding of a specific learning topic, encompassing both the prior conceptions the learner currently holds and the underlying reasons sustaining those conceptions. The framework characterizes students’ cognitive states along two tiers: an answer tier (what conception students hold) and a reasoning tier (why students hold that conception). Personalized resources are designed using a conflict-based strategy that directly presents discrepancies between students’ diagnosed cognitive states and scientifically accurate representations (Dong et al., 2023). Empirical research applying this framework has demonstrated significant positive effects on primary school mathematics achievement, with two-tier diagnosis-based personalized learning yielding greater achievement gains than both conventional instruction and non-personalized project-based learning (Lin et al., 2025). This finding aligns with the broader trend in recent conceptual change research: Pacaci et al. (2024), in a meta-analysis of conceptual change strategies in science education, reported that interventions targeting students’ prior misconceptions produce robust positive effects on learning outcomes.

The mechanisms through which personalized learning may produce its effects have been theoretically articulated and empirically examined from multiple perspectives. Kim (2012) emphasizes the role of affective and motivational factors in the design of effective personalized learning environments, arguing that personalization must address not only cognitive matching but also the maintenance of learners’ affective engagement with instruction. Building on this perspective, Plass and Pawar (2020) propose a dynamic framework in which effective personalization requires continuous adaptation to learners’ cognitive states and to moment-to-moment affective and motivational fluctuations, implying that the most consequential mechanisms operate at fine-grained temporal scales of the instructional process. Empirical evidence from intelligent tutoring systems supports this view: adaptive instruction that responds to learners’ affective states (e.g., confusion, engagement, boredom) has been shown to yield measurable improvements in learning outcomes (D’Mello et al., 2010; D’Mello, 2013). Within this context, the CVT framework offers a complementary account: by aligning instructional demands with learners’ existing competence, personalized learning may enhance students’ sense of control over learning tasks and may thereby support positive activating emotions such as enjoyment, curiosity, and surprise (Pekrun, 2006; Daniels and Stupnisky, 2012). These emotions can involve elevated arousal; however, elevated physiological activation is not specific to positive valence and may also accompany states such as confusion or anxiety. For conflict-based personalization, the direct presentation of discrepancies between students’ cognitive states and accurate representations may elicit an orienting response or other forms of sympathetic activation (Sequeira et al., 2009; Storbeck and Clore, 2008). Physiological arousal therefore provides one way to examine changes in activation during instruction, but EDA alone cannot determine the valence or psychological source of those changes.

Although substantial evidence has accumulated regarding the academic effects of personalized learning, existing research has primarily relied on outcome-level measurements—pre–post achievement gains, learning analytics indicators, or self-reported engagement—to evaluate its effectiveness. Comparatively little attention has been paid to moment-to-moment changes in physiological arousal during personalized instruction as a lesson unfolds. Examining these dynamics requires methods capable of capturing physiological activation at a fine temporal resolution, which motivates the present study’s use of EDA.

## 3 Methods

### 3.1 Research design

This study adopted a quasi-experimental between-subjects design, combined with continuous electrodermal activity (EDA) measurement throughout an entire classroom lesson, to compare students’ physiological arousal under personalized learning and conventional instruction conditions across three dimensions: overall trend, within-lesson temporal structure, and phase-specific differences.

The variables were defined as follows. The independent variable was instructional condition, dichotomously operationalized as personalized learning based on cognitive diagnosis and a conflict-based resource strategy (experimental group) and conventional instruction (control group). The two conditions covered the same curricular content and lesson duration, were taught by the same teacher in comparable regular classrooms, and differed principally in the planned learning approach used during the new-knowledge phase (see Section 3.3 for details).

The dependent variable was physiological arousal, assessed through the tonic and phasic components of EDA (Boucsein et al., 2012). Raw skin conductance (SC), expressed in microsiemens (μS), was used as the primary indicator of tonic arousal and as the principal scale for between-condition analyses. As a robustness check, the SC analyses were repeated using within-person min–max standardized skin conductance (SSC). SSC represents a student’s relative position within that student’s observed SC range and was not interpreted as an absolute between-person level. Skin conductance response frequency (SCRF) was used to represent phasic activation frequency. Because phasic responses may be influenced by movement and device use in authentic classrooms, the SCRF findings were interpreted cautiously and were not attributed exclusively to cognitive conflict. Learning outcome data (pre-/post-test scores) were reported separately as a supplementary learning outcome (see Section 4.4) and did not enter the core analytic framework for RQ1–RQ3.

Given that individual-level random assignment is infeasible in authentic school settings, the present study was conducted as a quasi-experiment using two intact classes as the assignment unit. Because only one intact class represented each condition, the design estimates condition-associated differences but cannot fully separate the instructional condition from other class-level influences. In addition, although the same teacher taught both classes, the teacher was not blinded to condition; therefore, expectancy-related differences in instructional delivery cannot be completely excluded.

### 3.2 Participants

Participants were 60 fourth-grade students from two intact classes at a primary school in Shanghai, China. Prior to the experiment, comparability between the two classes in terms of routine academic performance and classroom emotional states was assessed through teacher interviews, classroom observations, and questionnaire-based measurements. The two intact classes were randomly assigned at the class level to the experimental condition (initially *n* = 29, including 15 boys) and the control condition (initially *n* = 31, including 16 boys), with both classes exhibiting an approximately 1:1 gender ratio.

Of the 60 enrolled students, 50 were retained in the final EDA analytic sample (experimental class: *n* = 23; control class: *n* = 27). Three students were excluded for electrodermal non-responsiveness, operationally identified during signal-quality screening as an essentially flat lesson-long recording with no discernible electrodermal variation (experimental: *n* = 2; control: *n* = 1). Seven additional entire recordings were excluded because approximately 20% or more of the lesson consisted of missing, saturated, or otherwise unusable data (experimental: *n* = 4; control: *n* = 3). The data loss may have been related to instability or interference in wireless transmission when multiple devices were operating simultaneously; however, the precise technical cause could not be conclusively verified. The supplementary achievement-test dataset comprised all 60 enrolled students (experimental class: *n* = 29; control class: *n* = 31). The study was approved by Shanghai Normal University (approval issued as an official sealed document), and written informed consent was obtained from all participants and their legal guardians prior to data collection.

### 3.3 Instructional context and experimental procedure

The instructional content was “Introduction to Circles,” selected from Unit 5 (“Geometry in Practice”) of the first-semester fourth-grade primary mathematics curriculum. This content falls under the “Recognition of Shapes” strand within the broader “Shape and Geometry” domain, building upon students’ initial exposure to circular shapes in first grade. This content was selected because it is closely connected to students’ everyday experiences and supports the diagnosis and targeted revision of closely related geometric conceptions, including circle–sphere confusion.

The study procedure comprised two phases: instructional design and instructional implementation. During the instructional design phase, the research team iteratively developed a two-tier cognitive state diagnostic instrument (Lin et al., 2025), using data from over a thousand students across more than 30 classes in six public schools in Shanghai, to assess students’ cognitive states regarding the instructional content. The instrument characterized students’ cognitive states along two tiers simultaneously: an answer tier (what conception students hold) and a reasoning tier (why students hold that conception). Based on each student’s diagnostic profile, the research team developed personalized learning resources primarily using a conflict-based strategy: identifying the specific conceptual mechanism underlying each student’s cognitive state and designing targeted materials that presented discrepancies between the diagnosed conception and scientifically accurate representations. The conflict-based strategy was selected because it directly translated the answer- and reasoning-tier diagnoses into targeted personalized resources. For example, students whose diagnostic results indicated a “circle–sphere confusion” at the reasoning tier received learning resources that visually presented the spatial differences between the two, rendering the dimensional distinction immediately perceivable. The resources received by the experimental class therefore differed systematically in content from the uniform instructional materials used by the control class.

**Figure 1:**
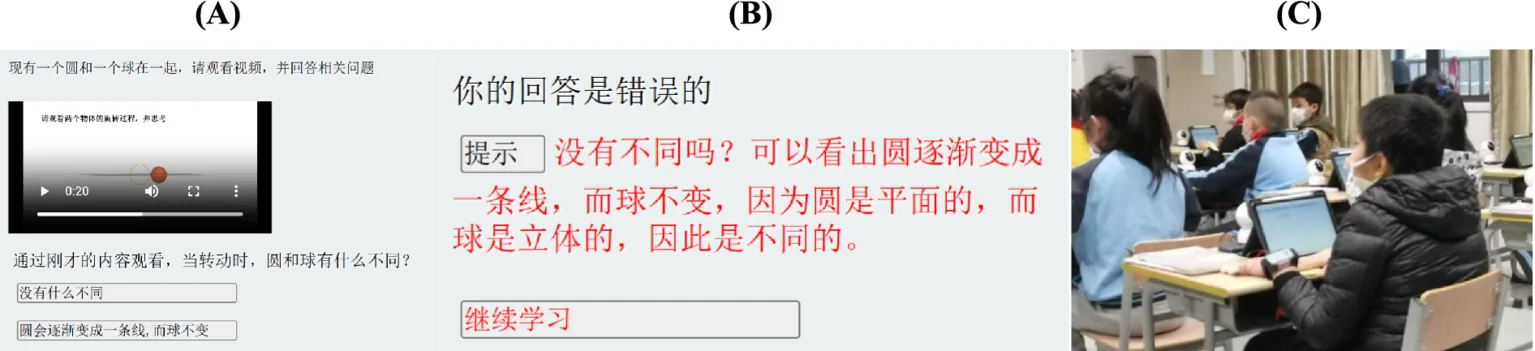
Conflict-based personalized-learning materials and classroom implementation. **(A)** A platform task presenting a circle–sphere comparison and prompting a student response; **(B)** Corrective feedback explaining the distinction between a circle and a sphere; **(C)** Students using the personalized-learning platform during the classroom lesson.

During the instructional implementation phase, all participants underwent a familiarization period with the EDA measurement device prior to formal instruction to reduce novelty-related arousal. The experimental class received personalized learning instruction, while the control class received conventional teacher-led instruction on the same content. Both classes were taught by the same experienced mathematics teacher to reduce variability attributable to differences between teachers. The teacher had 10 years of teaching experience, received a standardized briefing on the instructional procedures, and was asked to follow the pre-specified lesson plans while maintaining comparable instructional objectives, lesson duration, and general classroom management across the two classes. However, the teacher was aware of the instructional condition; therefore, unintentional differences in pacing, prompting, attention, or encouragement cannot be completely excluded. Instruction in the experimental class was supported by a personalized learning platform developed by the research team, as illustrated in Figure 1. The experimental class instruction included two personalized-learning episodes: the first occurred from 5.52 to 8.85 min and lasted 3.33 min; the second occurred from approximately 26.08 to 30.00 min and lasted approximately 3.92 min. The control class received teacher-led instruction during the corresponding clock-time intervals. For phase-based comparisons, data from the same clock-time intervals were used for both classes. Pre- and post-tests of academic achievement were administered immediately before and after the lesson, respectively.

### 3.4 Measures

#### 3.4.1 Academic achievement tests

The academic achievement tests were jointly developed by the research team and several experienced mathematics teachers and educational researchers. Both the pre-test and post-test consisted of 15 items, with a total score of 100 points. Internal consistency reliability was acceptable for both the pre-test (Cronbach’s *α* = 0.70) and the post-test (Cronbach’s *α* = 0.84). The two tests were designed to be comparable in item format, number, and difficulty.

#### 3.4.2 Physiological arousal measurement

EDA data were continuously collected throughout the lesson (approximately 35 min, covering the complete instructional process from introduction to summary) using a BIOPAC MP160 multi-channel physiological recording system at an initial sampling rate of 500 Hz. EDA electrodes were attached to the index and middle fingers of each participant’s non-dominant hand following standard placement protocols for electrodermal measurement (Boucsein et al., 2012). Students were instructed to minimize unnecessary movement of the monitored hand during recording.

Raw EDA data were preprocessed using AcqKnowledge 5.0 through the following steps: (1) downsampling to 10 Hz; (2) median smoothing with a window size of 10 samples to attenuate high-frequency noise; (3) applying a 1-Hz finite impulse response low-pass filter to preserve the slowly varying signal while further attenuating residual noise; and (4) linearly interpolating isolated brief breakpoints by connecting the nearest valid sample immediately before and after each gap.

Mean raw skin conductance (SC) within each analysis window, expressed in microsiemens (μS), was used as the primary indicator of tonic physiological arousal. As a robustness check, each participant’s retained SC recording was also transformed using within-person min–max normalization to produce standardized skin conductance (SSC), with values ranging from 0 to 1. SSC represents a student’s relative position within that student’s observed SC range and was not interpreted as an absolute between-person level. Skin conductance response frequency (SCRF) was extracted using Ledalab 3.49 and defined as the number of discrete skin conductance responses exceeding the 0.05 μS threshold per minute. SCRF was retained as an indicator of phasic activation frequency. Because phasic responses may be influenced by movement and device interaction in authentic classrooms, SCRF was interpreted cautiously and was not treated as direct evidence of a specific emotion or cognitive conflict. No additional manual peak removal was performed.

#### 3.4.3 Annotation of instructional events

Classroom video was recorded synchronously throughout the lesson. Drawing on the teacher’s instructional design documents and video playback, the research team annotated the type of instructional activity (e.g., introduction, lecture, personalized learning, practice, summary) according to a predefined coding scheme. The resulting time stamps were used to define four adjacent-window contrasts for RQ2 and to compare the two classes over the same clock-time windows. The synchronized video was used for instructional-event annotation, not for peak-by-peak artifact coding. Because the fixed, non-overlapping two-minute windows did not coincide exactly with every instructional event boundary, the results were interpreted as changes associated with the start or end of an instructional episode rather than as instantaneous physiological responses to the event.

### 3.5 Data analysis

The analytic strategy addressed the three research questions at the levels of overall directional trend, within-lesson temporal structure, and phase-specific between-group differences. Individual repeated observations were retained in linear mixed-effects models to account for within-student dependence. Raw skin conductance (SC) was used for the primary analyses, and the corresponding analyses were repeated using within-person min–max standardized skin conductance (SSC) as a robustness check.

**Figure 2:**
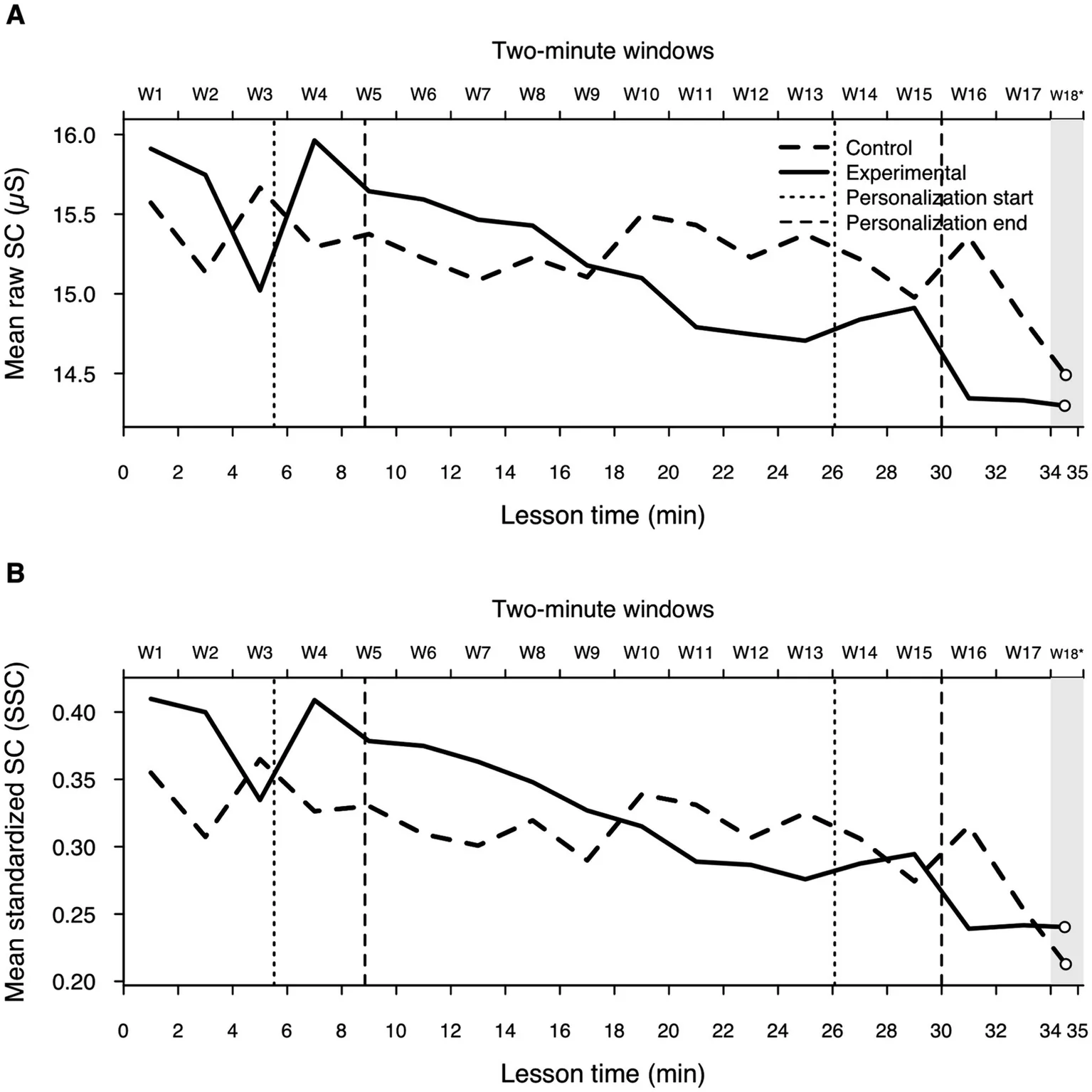
Group-mean raw skin conductance (SC) and within-person min–max standardized skin conductance (SSC) across the lesson. **(A)** shows group-mean raw SC, and **(B)** shows group-mean within-person min–max SSC. In both panels, the dashed and solid trajectories represent the control and experimental classes, respectively. W1–W17 denote consecutive non-overlapping 2-minute intervals from 0–2 to 32–34 min. Dotted vertical lines mark the starts of the two personalized-learning episodes (5.52 and 26.08 min), and dashed vertical lines mark their ends (8.85 and approximately 30 min). The control class received conventional instruction during the corresponding clock-time intervals. W18 represents the incomplete final interval and is shown for descriptive purposes only; all inferential analyses were based on W1–W17.

Signal quality was checked for each student and each two-minute window. At the recording level, entire recordings with approximately 20% or more missing, saturated, or otherwise unusable data were excluded before window aggregation. Within retained recordings, windows containing more than 30 s of invalid signals due to motion artifacts or signal saturation were treated as missing. The lesson yielded 17 complete non-overlapping two-minute windows from 0 to 34 min; the final incomplete fragment was excluded from inferential analyses but retained in Figure 2 for descriptive visualization.

#### 3.5.1 Overall directional trend (RQ1)

To characterize the overall direction of physiological arousal across the lesson, Mann–Kendall trend tests were conducted separately for the experimental and control classes using their group-mean SSC values across the 17 complete two-minute windows. These windows were consecutive and non-overlapping: W1 covered 0–2 min, W2 covered 2–4 min, and so forth, with W17 covering 32–34 min. The residual segment after 34 min was labeled W18 and retained only in Figure 2 for descriptive visualization; it was excluded from all window-based inferential analyses. This nonparametric test identifies monotonic trends without assuming a specific trajectory form. Because the standard Mann–Kendall test does not model serial dependence, it was treated as a complementary trend summary; primary individual-level inference was based on the AR (1) mixed-effects models.

Linear mixed-effects models were then fitted to individual raw-SC observations aggregated into two-, four-, and six-minute windows. Each model included condition, centered time, and Condition × Time as fixed effects, with a student random intercept and a first-order autoregressive [AR (1)] residual structure. Time was measured in minutes and centered at 17 min; the experimental class served as the reference group for the reported condition-specific slopes. Final fixed-effect estimates were obtained using restricted maximum likelihood, whereas the linear and quadratic models were compared using maximum likelihood. A quadratic time term was evaluated by likelihood-ratio comparison. The two-minute analysis included 17 complete windows covering 0–34 min, the four-minute analysis included eight complete windows covering 0–32 min, and the six-minute analysis included five complete windows covering 0–30 min. Two-minute windows were used as the primary resolution because they retained temporal detail in relation to the two personalized-learning episodes, whereas four- and six-minute windows were used as robustness checks. This selection reflected the trade-off between temporal resolution and measurement stability discussed by Leiner et al. (2012).

#### 3.5.2 Within-lesson temporal structure (RQ2)

To examine the within-lesson temporal structure in relation to the documented instructional transitions, four event-boundary adjacent-window comparisons were defined from the instructional timeline: the start and end of the first personalized-learning episode, documented at 5.52 and 8.85 min, respectively (W3-W4: 4–6 to 6–8 min; W4-W5: 6–8 to 8–10 min), and the start and end of the second episode, documented at 26.08 and approximately 30 min, respectively (W13-W14: 24–26 to 26–28 min; W15-W16: 28–30 to 30–32 min). The event-boundary analysis used the two-minute resolution because the episodes lasted approximately 3.33 and 3.92 min; four- or six-minute windows would combine each episode with a substantial portion of the surrounding instructional phases. For each comparison, a linear mixed-effects model included condition, phase, and Condition × Phase as fixed effects and a student random intercept. The interaction tested whether the two classes changed differently across the same adjacent windows. The four interaction tests were adjusted using the Holm procedure. The analyses were conducted first with raw SC and then repeated with SSC as a robustness check.

#### 3.5.3 Phase-specific between-group differences (RQ3)

For each student, raw SC and SCRF were summarized over the two personalized-learning intervals. Data from the same clock-time intervals were extracted for the control class, ensuring matched instructional durations. Independent-samples *t*-tests were used to compare raw SC and SCRF between the two classes, and the SC comparison was repeated using SSC as a robustness check. Nonsignificant differences were not interpreted as evidence of equivalence.

Mann–Kendall tests and descriptive analyses were conducted using IBM SPSS Statistics 27, and linear mixed-effects models were fitted in R 4.6.1 using the nlme package (version 3.1-169). All tests were two-sided with *α* = 0.05. Cohen’s *d* was reported for independent-samples comparisons, and Holm-adjusted *p*-values were reported for the four adjacent-window interactions.

## 4 Results

### 4.1 RQ1: temporal trend of physiological arousal across the lesson

To examine whether physiological arousal exhibited a directional trend across the lesson, Mann–Kendall tests were applied separately to the group-mean SSC series across 17 complete two-minute windows. Both classes showed significant monotonic declines. For the control class, *Z* = −2.266, *p* = 0.023, with a Sen’s slope of −0.00167 SSC units/min; for the experimental class, *Z* = −4.490, *p* < 0.001, with a Sen’s slope of −0.00552 SSC units/min (Table 1).

**Table 1:** Mann–Kendall trend tests of group-mean SSC across 17 complete two-minute windows.

Group	Number of windows	*Z*	*p*	Sen’s slope (SSC/min)
Control	17	−2.266	0.023	−0.00167
Experimental	17	−4.490	<0.001	−0.00552

Linear mixed-effects models based on individual raw-SC observations further showed that the experimental-class slope was significantly negative at the two-, four-, and six-minute resolutions (Table 2). At the two-minute resolution, the control-minus-experimental difference in slope was +0.0322 μS/min (SE = 0.0121, *p* = 0.008). The corresponding differences were +0.0362 μS/min at 4 min (SE = 0.0216, *p* = 0.094) and +0.0206 μS/min at 6 min (SE = 0.0179, *p* = 0.251). The SSC robustness analyses also produced inconsistent interaction results across window lengths. In addition, quadratic time terms did not improve model fit at any resolution. These results support an overall declining lesson-level trend, whereas the evidence for a consistent between-condition difference in the rate of decline was dependent on window length and should therefore be interpreted cautiously.

**Table 2:** Linear mixed-effects models of individual SC trends by window length.

Outcome	Window	Experimental slope/min	Slope *p*	Condition × Time estimate (SE); *p*	Quadratic LRT *p*
Raw SC (μS)	2 min	−0.0466	<0.001	+0.0322 (0.0121); 0.008	0.849
Raw SC (μS)	4 min	−0.0429	0.007	+0.0362 (0.0216); 0.094	0.929
Raw SC (μS)	6 min	−0.0315	0.018	+0.0206 (0.0179); 0.251	0.584
SSC	2 min	−0.00518	<0.001	+0.00282 (0.00145); 0.052	0.824
SSC	4 min	−0.00497	<0.001	+0.00369 (0.00167); 0.028	0.873
SSC	6 min	−0.00409	0.002	+0.00247 (0.00179); 0.170	0.752

The experimental class was the reference group. Condition × Time estimates are the control-minus-experimental differences in linear slope; each estimate is followed by its standard error in parentheses and *p*-value. LRT, likelihood-ratio test.

**Figure 3:**
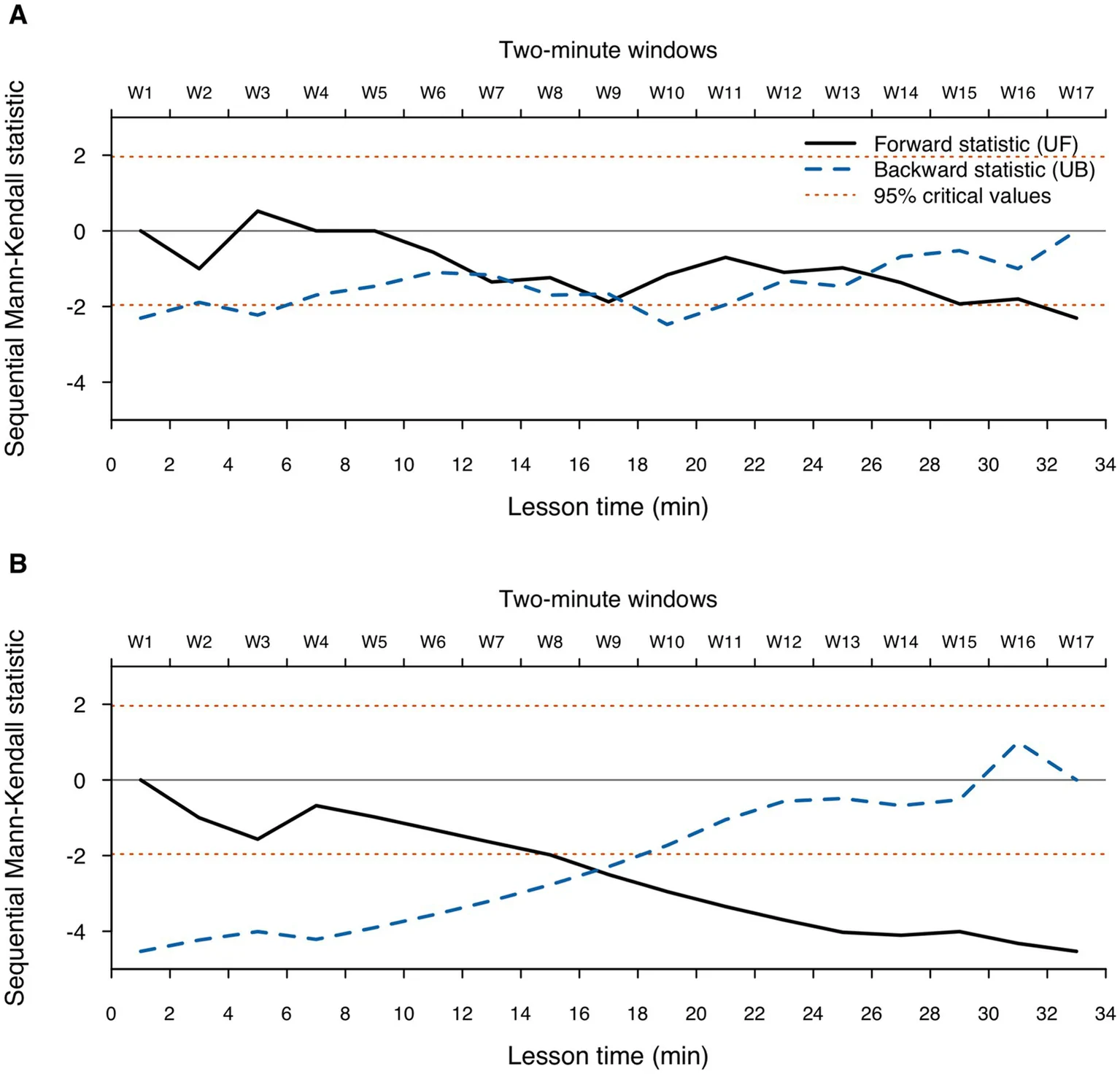
Sequential Mann–Kendall plots of group-mean SSC across 17 complete two-minute windows. The upper and lower panels represent the control and experimental classes, respectively. The solid curve represents the forward statistic (UF), the dashed curve represents the backward statistic (UB), the horizontal solid line indicates zero, and the dotted horizontal lines indicate the two-sided critical values of ±1.96. The plots describe cumulative trend statistics and are not interpreted as additional change-point tests. **(A)** Control class. **(B)** Experimental class.

Figure 3 presents the sequential Mann–Kendall statistics as a descriptive visualization of the SSC trends. In both panels, the forward statistic moved into the negative region and crossed the lower critical boundary, consistent with the declining trends reported in Table 1.

### 4.2 RQ2: dynamic trajectory of physiological arousal

Four event-boundary adjacent-window comparisons, defined from the documented instructional timeline, were used to examine whether the two classes changed differently around the starts and ends of the two personalized-learning episodes. At the first start-associated comparison, W3 (4–6 min) versus W4 (6–8 min), the raw-SC Condition × Phase interaction was significant, *F* (1, 48) = 9.12, unadjusted *p* = 0.004, Holm-adjusted *p* = 0.012. Raw SC increased by 0.942 μS in the experimental class and decreased by 0.372 μS in the control class, yielding an experimental-minus-control difference in change of +1.314 μS. The corresponding SSC interaction was also significant, *F* (1, 48) = 9.09, Holm-adjusted *p* = 0.012.

At the second end-associated comparison, W15 (28–30 min) versus W16 (30–32 min), the raw-SC Condition × Phase interaction was significant, *F* (1, 48) = 14.55, *p* < 0.001, Holm-adjusted *p* = 0.002. Raw SC decreased by 0.568 μS in the experimental class and increased by 0.378 μS in the control class, yielding a difference in change of −0.946 μS. The same pattern was found in the SSC robustness analysis, *F* (1, 48) = 16.05, Holm-adjusted *p* = 0.001. The first end-associated and second start-associated interactions were not significant after Holm adjustment on either scale (Table 3).

**Table 3:** Condition × phase interactions for the four event-boundary adjacent-window comparisons.

Transition	Windows	Raw SC change difference; *F*	Raw Holm *p*	SSC change difference; *F*	SSC Holm *p*
First start-associated	W3-W4	+1.314 μS; 9.12	0.012	+0.113; 9.09	0.012
First end-associated	W4-W5	−0.400 μS; 2.99	0.180	−0.034; 1.74	0.387
Second start-associated	W13-W14	+0.288 μS; 1.38	0.246	+0.031; 1.17	0.387
Second end-associated	W15-W16	−0.946 μS; 14.55	0.002	−0.096; 16.05	0.001

Change difference = (later window − earlier window) in the experimental class minus the corresponding change in the control class. All interaction tests have d*f* = (1, 48). Holm-adjusted *p*-values account for the four comparisons separately for raw SC and SSC.

Because the instructional boundaries did not coincide exactly with all two-minute window boundaries, these results are interpreted as transition-associated changes rather than instantaneous event responses. Figure 2 provides descriptive visualizations of the raw-SC and SSC trajectories across the lesson.

### 4.3 RQ3: differences in physiological arousal level and activation frequency during the critical instructional phase

Phase-average raw SC was compared between the two classes over the same two clock-time intervals corresponding to the personalized-learning episodes (5.52–8.85 min and 26.08–30.00 min). No significant difference was found. The experimental class had a mean raw SC of 15.370 μS (SD = 4.292), and the control class had a mean of 15.219 μS (SD = 4.150), *t* (48) = 0.127, *p* = 0.900, Cohen’s *d* = 0.04. The experimental-minus-control mean difference was 0.151 μS, 95% CI [−2.254, 2.556].

For activation frequency, SCRF was significantly higher in the experimental class (*M* = 5.381, SD = 0.948) than in the control class (*M* = 2.753, SD = 0.955), *t* (48) = 9.728, *p* < 0.001, Cohen’s *d* = 2.76. The experimental-minus-control mean difference was 2.628 responses/min, 95% CI [2.085, 3.171]. This result indicates a higher frequency of detected skin conductance responses during the experimental instructional phase; it is not interpreted as direct evidence of cognitive conflict or a particular emotional state (see Table 4).

**Table 4:** Phase-specific comparisons of physiological arousal between conditions.

Outcome	Control *M* (SD)	Experimental *M* (SD)	*t*(48)	*p*	Exp. − Ctrl.	95% CI	*d* [95% CI]
Raw SC (μS)	15.219 (4.150)	15.370 (4.292)	0.127	0.900	0.151	[−2.254, 2.556]	0.04 [−0.52, 0.59]
Min–max SSC	0.309 (0.072)	0.346 (0.108)	1.419	0.162	0.036	[−0.015, 0.088]	0.40 [−0.16, 0.96]
SCRF (responses/min)	2.753 (0.955)	5.381 (0.948)	9.728	<0.001	2.628	[2.085, 3.171]	2.76 [1.97, 3.54]

*M*, mean; SD, standard deviation; CI, confidence interval. Raw SC is the primary measure of tonic arousal; within-person min–max SSC is used as a robustness check. SCRF represents the frequency of detected skin conductance responses. Mean differences and *t*-values are calculated as experimental minus control. The penultimate column reports 95% CIs for the mean difference, and the final column reports Cohen’s *d* with its 95% CI.

### 4.4 Supplementary finding: academic achievement

Academic achievement was analyzed separately as a supplementary learning outcome. The achievement-test dataset included the 60 enrolled students (experimental class: *n* = 29; control class: *n* = 31). Before instruction, mathematics scores did not differ significantly between the two classes (experimental: *M* = 34.34, SD = 13.11; control: *M* = 34.10, SD = 12.27), *t* (58) = 0.074, *p* = 0.941. After instruction, the experimental class scored significantly higher than the control class (experimental: *M* = 80.10, SD = 14.34; control: *M* = 68.56, SD = 16.52), *t* (58) = 2.898, *p* = 0.005, Cohen’s *d* = 0.76, with an experimental-minus-control mean difference of 11.54 points (Table 5).

**Table 5:** Descriptive statistics and independent-samples t-tests for mathematics achievement.

Measurement	Control *M* (SD)	Experimental *M* (SD)	*t* (d*f*)	*p*	MD (Exp. − Ctrl.)	95% CI of MD	Cohen’s *d*
Pre-test	34.10 (12.27)	34.34 (13.11)	0.07 (58)	0.941	0.245	[−6.39, 6.88]	0.02
Post-test	68.56 (16.52)	80.10 (14.34)	2.90 (58)	0.005	11.555	[3.51, 19.60]	0.76

*M*, mean; SD, standard deviation; MD, mean difference; CI, confidence interval. Mean differences and *t*-values are calculated as experimental minus control.

## 5 Discussion

### 5.1 Lesson-wide decline in physiological arousal

The group-mean SSC series declined significantly across the lesson in both classes, and the experimental-class raw-SC slope remained significantly negative at the two-, four-, and six-minute resolutions. This lesson-wide decline is consistent with previous observations of attenuating physiological activation during sustained classroom activity and with theoretical accounts of limits on sustained attention (Yu et al., 2024; Sharpe and Tyndall, 2025). However, the decline should not be interpreted as a direct measure of decreasing engagement, because SC indicates sympathetic activation but cannot identify its specific cognitive, affective, or behavioral source.

The Condition × Time interaction was significant in the primary two-minute raw-SC model but not in the four- or six-minute raw-SC models. The SSC robustness analyses likewise produced inconsistent interaction results across window lengths. Together, these findings support a general lesson-wide decline in both classes, but they do not demonstrate a consistent between-condition difference in the overall rate of decline across the three temporal resolutions examined.

### 5.2 Condition-associated differences in SC changes at instructional transitions

Two of the four event-boundary adjacent-window comparisons remained significant after Holm correction for both raw SC and SSC. At the start of the first personalized-learning episode, SC increased in the experimental class while decreasing in the control class over the same clock-time transition. At the documented end of the second episode, SC decreased in the experimental class while increasing in the control class. The significant Condition × Phase interactions therefore indicate localized differences in SC change between the two classes at these two instructional transitions, rather than changes established only within the experimental class.

One possible interpretation is that the first start-associated change was related to the conflict-based features of the personalized resources. Presenting discrepancies between students’ diagnosed cognitive states and scientifically accurate representations may elicit orienting or effort-related physiological activation (Sequeira et al., 2009; Bouzidi and Gendolla, 2023). However, the present findings do not establish that cognitive conflict caused the observed SC change. The two-minute windows were only approximately aligned with the instructional boundaries, and EDA alone cannot determine emotional valence or distinguish cognitive conflict from other sources of sympathetic activation, such as attention, uncertainty, anxiety, movement, or device interaction. Similarly, the decline associated with the end of the second episode may reflect reduced activation following the activity, but it cannot be interpreted as direct evidence that cognitive conflict was resolved or that arousal returned to baseline.

### 5.3 Higher SCRF without a detected difference in phase-average SC

The phase-specific SCRF comparison showed that the experimental class had a significantly higher frequency of detected skin conductance responses than the control class (*M* = 5.381 vs. 2.753 responses/min, *p* < 0.001, Cohen’s *d* = 2.76). In contrast, the matched-time comparison found no significant between-class difference in phase-average raw SC (*p* = 0.900), and the SSC robustness analysis produced the same nonsignificant conclusion (*p* = 0.162). These findings indicate a higher detected response frequency in the experimental class alongside no detected difference in phase-average tonic SC; however, the nonsignificant SC results do not establish physiological equivalence between the classes.

The nonsignificant phase-average comparison does not imply that the within-phase trajectories were identical. As illustrated in Figure 2, against the general lesson-wide decline, the experimental-class raw-SC and SSC trajectories rose around the beginning of the first personalized-learning episode and temporarily flattened or increased during the second episode, whereas the control-class trajectory generally continued to decline over the corresponding windows. This descriptive pattern suggests that averaging across the complete instructional intervals may obscure localized attenuation or temporary reversal of the overall decline. The trajectories are descriptive; formal inferential support is limited to the Condition × Phase interactions reported in Section 4.2.

SCRF represents the frequency of discrete phasic skin conductance responses, whereas phase-average SC characterizes the more slowly varying tonic level of sympathetic activation (Boucsein et al., 2012). The observed pattern is compatible with more frequent transient activation during the experimental instructional activities and resembles the event-related EDA responses reported in adaptive-learning feedback research (Horvers et al., 2024). However, it does not establish that personalized learning or cognitive conflict caused the higher SCRF. In an active classroom, detected responses may also be influenced by hand movement, platform manipulation, visual attention, and response behavior. The SCRF difference should therefore be interpreted as a condition-associated difference in detected activation frequency rather than as direct evidence of cognitive conflict, academic emotion, or positive engagement.

### 5.4 Parallel physiological and achievement findings

The experimental class achieved significantly higher post-test mathematics scores than the control class, consistent with research suggesting that instruction targeting students’ prior conceptions can support conceptual change and learning (Pacaci et al., 2024; Lin et al., 2025). This achievement difference co-occurred at the class level with condition-associated SC changes at two instructional transitions and a higher SCRF in the experimental class. These findings provide parallel information about learning performance and physiological activation, but they do not establish a relationship between the two outcomes.

The physiological and achievement results were obtained from separate analyses and partly different analytic samples, and no individual-level association or mediation analysis was conducted. Higher achievement therefore cannot demonstrate that the observed SC changes or higher SCRF were beneficial, positively valenced, or responsible for the learning difference. Nevertheless, their co-occurrence provides a basis for future research to test whether event-associated physiological dynamics are related to conceptual change or learning gains using repeated interventions, individual-level association analyses, and explicit mediation designs.

### 5.5 Implications

This study offers empirical and methodological implications for research on physiological arousal during classroom learning. Empirically, the findings show that lesson-wide decline, localized SC changes, phase-average tonic activation, and SCRF can provide different information about physiological arousal. This extends the analysis beyond static phase averages and illustrates the value of examining both global trajectories and localized changes when investigating the physiological arousal dimension of academic emotion.

Methodologically, the study demonstrates an event-oriented approach to continuous classroom EDA by retaining individual repeated observations and comparing the two classes over the same instructional transitions. Using instructional time stamps to define event-boundary comparisons makes it possible to examine whether localized changes differ between conditions without relying solely on within-group *post hoc* comparisons. This approach may be useful in classroom studies in which instructional events can be reliably documented.

Continuous EDA may also provide complementary process information for the design and evaluation of personalized-learning environments. However, EDA and SCRF should not be treated as direct indicators of engagement, cognitive conflict, or emotional valence. Future adaptive applications should combine physiological signals with behavioral observations, platform-interaction data, and self-report measures. The present findings apply specifically to the cognitive-diagnostic, conflict-based form of personalized learning examined here and should not be generalized directly to other forms of personalized learning.

## 6 Conclusion

### 6.1 Main findings

This quasi-experimental study combined continuous EDA recording with time-resolved analyses to examine students’ physiological arousal under a cognitive-diagnostic, conflict-based personalized-learning condition and conventional instruction. The EDA analytic sample comprised 50 students from two intact fourth-grade classes. Raw SC was used as the primary measure of tonic activation, within-person min–max SSC was used for robustness analyses, and SCRF was used to characterize activation frequency.

Three main findings emerged. First, group-mean SSC declined significantly across the lesson in both classes, and the experimental-class raw-SC slope remained negative at the two-, four-, and six-minute resolutions. However, the Condition × Time interaction was not consistent across these resolutions, so a stable between-condition difference in the overall decline rate was not demonstrated.

Second, two of the four event-boundary Condition × Phase interactions remained significant after Holm correction on both the raw-SC and SSC scales. The two classes changed differently at the first personalized-learning start and the second personalized-learning end, indicating localized differences in SC change at these instructional transitions.

Third, matched-time phase-average raw SC did not differ significantly between the classes, and the SSC robustness analysis produced the same nonsignificant conclusion. However, the experimental class showed a significantly higher phase-specific SCRF than the control class, indicating a higher frequency of detected skin conductance responses. The experimental class also achieved higher post-test mathematics scores, although the relationship between the physiological findings and achievement was not directly tested.

Taken together, the findings indicate that the personalized-learning condition examined here was associated with localized SC differences at two instructional transitions and a higher detected response frequency within a generally declining lesson-wide trajectory, but not with a consistently different global decline rate or phase-average tonic SC level. These findings are specific to the cognitive-diagnostic, conflict-based form of personalized learning examined in this study and do not establish a particular emotional valence, cognitive-conflict mechanism, or causal pathway to achievement.

### 6.2 Limitations and future directions

Several limitations delimit these findings. The EDA analysis involved one experimental and one control class from a single school, and instructional condition was therefore confounded with class membership. Individual random assignment was not feasible, and the teacher delivered both conditions without blinding. Consequently, class-level characteristics and expectancy-related differences in pacing, prompting, attention, or encouragement cannot be completely excluded. Of the 60 enrolled students, 50 were included in the final EDA analysis; six experimental-class and four control-class students were excluded because they did not provide usable complete recordings or showed persistent signal-quality problems. These features limit causal inference, statistical precision, and generalizability.

EDA reflects sympathetic activation but cannot determine its specific psychological source or emotional valence. The study did not include event-contingent self-reports or other convergent affective measures, and synchronized behavioral coding was not available to separate physiological responses from movement, platform interaction, visual orienting, and response behavior. SCRF may be particularly affected by these factors. In addition, the fixed two-minute windows only approximately coincided with the instructional-event boundaries. The observed changes should therefore be understood as transition-associated patterns rather than instantaneous responses to specific events.

Future research should replicate the study across multiple schools, classes, teachers, subject areas, and age groups and, where feasible, use class-level randomization or counterbalanced designs. Combining EDA with synchronized movement coding, platform-interaction logs, behavioral observations, and event-level self-reports would help distinguish the sources and emotional valence of physiological activation. Future studies could also use repeated interventions and individual-level association or mediation analyses to examine whether physiological dynamics are related to conceptual change and learning outcomes.

## Data Availability

The raw data supporting the conclusions of this article are not publicly available because they contain data from minor participants and are subject to ethical and privacy restrictions. De-identified data may be available from the corresponding author upon reasonable request, subject to applicable ethical and institutional requirements.
